# A245 THE ROLE OF FODMAP-MICROBIOTA INTERACTIONS IN IBS-RELATED VISCERAL PAIN

**DOI:** 10.1093/jcag/gwae059.245

**Published:** 2025-02-10

**Authors:** S E Ables, A S Bennett, S J Vanner, A E Lomax, D E Reed

**Affiliations:** Queen’s University, Kingston, ON, Canada; Queen’s University, Kingston, ON, Canada; Queen’s University, Kingston, ON, Canada; Queen’s University, Kingston, ON, Canada; Queen’s University, Kingston, ON, Canada

## Abstract

**Background:**

Irritable bowel syndrome (IBS) is characterized by abdominal pain and altered bowel habits. IBS is more common in females. Furthermore, food and stress are two common triggers of abdominal pain in IBS patients. A low FODMAP diet (LFD) reduces abdominal pain in a subgroup of IBS patients, although the mechanism behind this is unclear. We hypothesize that a LFD improves abdominal pain by altering neuroactive gut luminal mediators.

**Aims:**

1. Explore the effect of a LFD on IBS symptoms and gut luminal mediator-induced neuronal activity.

2. Investigate whether stress alters IBS symptoms and luminal mediator-induced neuronal activity.

3. Examine sex differences in dorsal root ganglion (DRG) neuron sensitivity to luminal mediators.

**Methods:**

Six female IBS patients followed a LFD for six weeks. Participants donated stool samples and completed the IBS Symptom Severity Scale (IBS-SSS) and the Depression, Anxiety, and Stress Scale (DASS-21) questionnaires. Male and female C57Bl6 mouse DRG neurons were incubated with IBS patient and healthy control (HC) fecal supernatant (FS). Capsaicin-induced Ca^2+^ influx was quantified to measure neuronal activity. N refers to number of mice.

**Results:**

Overall, IBS FS collected prior to the LFD caused a 17% increase in neuronal activity compared to HC FS (N=32 mice, p<0.05). This finding was sex dependent, as only DRG neurons from female mice exhibited an IBS FS-induced increase in neuronal activity (N=18, p<0.005). There was no difference in neuronal activity caused by IBS FS versus HC FS in neurons from male mice (N=16, p>0.05). FS collected from patients after a LFD did not alter neuronal activity compared to FS collected before the LFD (N=32, p>0.05). However, when solely analyzing neurons from female mice, LFD FS-induced neuronal activity was lower than IBS FS-treated neuronal activity (N=16, p<0.05).

Four IBS patients reported a clinically significant improvement in symptoms (>50-point reduction in IBS-SSS scores) during the LFD. Of these, two had a >50% reduction in IBS-SSS scores and their LFD FS reduced neuronal activity compared to their IBS FS (N=10, p<0.05). The two others had modest symptom improvement on the LFD; their LFD FS had similar effect on neuronal activity compared to their IBS FS (N=11, p>0.05). Additionally, in two IBS patients, periods of severe stress (DASS-21 stress sub-scale score >26) corresponded to high IBS-SSS scores; FS from these time points increased neuronal activity (N=10, p<0.05).

**Conclusions:**

In a subgroup of IBS patients, a LFD improves symptoms and reduces the excitatory effects of luminal mediators on sensory neuronal activation. Stress appears to increase the excitatory effects of luminal mediators and exacerbates IBS symptoms. Finally, neurons from female mice may be more sensitive to pro-nociceptive luminal mediators, potentially contributing to abdominal pain in female IBS patients.

**Funding Agencies:**

CIHR

